# Can the Geriatric Day Hospital Act As a Hub for Services for Older People across the Spectrum of Ageing from Active Ageing to Advanced Frailty?

**DOI:** 10.3389/fmed.2018.00023

**Published:** 2018-03-02

**Authors:** Rónán O’Caoimh, Siobhán Kennelly, Diamuid O’Shea

**Affiliations:** ^1^Clinical Sciences Institute, National University of Ireland, Galway, Galway, Ireland; ^2^National Clinical Programme for Older People, Royal College of Physicians of Ireland, Dublin, Ireland; ^3^Department of Medicine for the Elderly, Connolly Hospital, Blanchardstown, Dublin, Ireland; ^4^Department of Geriatric Medicine, St Vincent’s University Hospital, Dublin, Ireland

**Keywords:** Day Hospital, frailty, pre-frailty, Comprehensive Geriatric Assessment, geriatrics, ageing

This article examines the potential of the Geriatric Day Hospital to address the challenge of an aging society, which has begun to place an emphasis on the promotion of active and healthy aging, yet faces rising numbers of pre-frail and frail older adults with complex care needs. Can the Day Hospital model become a hub for the care of older adults across the spectrum of aging? This article explores its origins and traditional role in delivering Comprehensive Geriatric Assessment (CGA), assesses how it is currently being used to identify, triage and manage frailty, providing reablement, chronic disease management and anticipatory care planning, and discusses future models focused on the prevention, surveillance, and monitoring of frailty. It will examine how such approaches could increasingly deploy information communication technologies (ICT) using the Geriatric Day Hospital as a hub to maintain older adults in their home environment, to promote active aging, while both preventing and managing frailty.

A consequence of population aging worldwide, but particularly in the European Union (1), is high rates of frailty (2) and multi-morbidity (3) among older people. This has directed health polices toward prevention and the pursuit of active and healthy aging (4, 5) but has nevertheless resulted in increasing numbers of older patients who could benefit from specialist geriatric services, meaning that limited resources must be stretched further. A fundamental challenge now faced in the management of older adults with multiple interacting medical and social problems is how to move from a single system, unidimensional construct to a more holistic and multidimensional model of care (6) that promotes preventative approaches and reablement as well as providing long-term management and rehabilitation. In this evolving environment, established ambulatory models of care for older people with complex needs that traditionally focused on the latter such as the Geriatric Day Hospital could be leveraged to find an additional purpose, to promote active and healthy aging and manage pre-frailty, while continuing to support the care of frail older adults.

The Geriatric Day Hospital, which originated in the United Kingdom in the 1950s, is a dedicated outpatient service providing specialized, interdisciplinary, ambulatory, and usually rapid access geriatric medical, nursing, and rehabilitation care to community-dwelling older patients, whose primary strength is arguably the flexibility it offers (7). Day Hospitals represented an evolution in primary and secondary level ambulatory care models for older people with complex needs. Those attending Day Hospitals receive and benefit from CGA, individualized multi-domain assessment by a multidisciplinary team using validated scales and interventions that reduce adverse outcomes, hospital admission, and length of hospitalization (8). CGA is, however, labor intensive and economically costly; the Day Hospital rationalizes and targets this limited resource “under one roof” (9) in an effective (10) and cost-effective manner (11). A recent systematic review summarizing evidence from 16 studies comparing Geriatric Day Hospitals to non-integrated, non-comprehensive services suggests that it is superior, reducing the risk of functional impairment, institutionalization and death, albeit the evidence remains limited, and no cost benefit has been established (9). Further, there is much heterogeneity in terms of what is offered and to whom with studies varying in their sampling strategies; the strongest evidence being for models focusing on geriatric rehabilitation and subspecialty diseases such as stroke, dementia, and heart failure (12). There is limited evidence for its use in prevention and health promotion.

The most recent change in Day Hospitals is a shift toward specialty services, clinics, and ambulatory investigations. Paralleling this change, the relatively new construct of frailty has begun to replace historical models of geriatric care and is increasingly being used in Day Hospitals to select and risk-stratify attendees. Frailty is a multi-factorial state correlating with vulnerability, disability, comorbidity, and self-reported health status with a recognized prodrome, pre-frailty (13). This construct recognizes that the stereotypical characteristics of community-dwelling older patients such as age are insufficient to identify older adults deemed most at risk of adverse healthcare outcomes and hence most in need of CGA (14). Given the current ageing demographic (1), the construct of frailty can help to identify those most likely to benefit from the Day Hospital (15). However, few studies have been conducted to examine the role of the Day Hospital in identifying frail older adults. A study using the SHARE Frailty Index to examine the prevalence of frailty among community-dwelling attendees at a University Hospital affiliated Day Hospital in Ireland found that the prevalence of frailty in this transitional care sample was high at 32% (16). In France, another observational cross sectional study applying consecutive sampling using Fried’s criteria, to a similar sample referred to a single geriatric unit, found a higher prevalence of 51% (17). Levels of pre-frailty were also high in both samples at 26 and 41%, respectively. These data represent values between those in community and inpatient settings (2, 18, 19), suggesting that most attendees at Day Hospitals have high-care requirements, but also represent an ideal population to target for measures designed to tackle, prevent, and reverse frailty, including those that promote active and health aging at population-level.

The Geriatric Day Hospital is also increasingly being used as a coordination center to deliver integrated care (20) between acute services (emergency departments, acute medicine assessment units, and inpatient wards) (21), rehabilitation services (formal inpatient and early supported discharge teams), community services (primary care teams and general practitioners), and public health services designed to promote active and healthy aging in place, the person’s own community (implementation of local, national, and transnational population-level preventative healthcare strategies). A consistent approach to identify frailty across primary, secondary, and social care, e.g., coordinated by case managers, community public health nurses, or primary care physicians can promote equity of access to CGA services (22). Identifying pre-frailty and frailty in people attending for Day Hospital assessment services may be looked on as a form of case finding and an opportunity for health promotion (23). This, in turn, allows for comprehensive proactive management of conditions that result in high levels of acute care episodes. Models that reflect mutual goal setting in determining outcomes (e.g., Goal Attainment Scores) will be increasingly used and will provide a key element of person-centered support in the Day Hospital (20). Screening for complexity and pre-frailty also may have the additional benefit of taking on a much more proactive approach to the planning of care needs and potentially impact on transitions of care given that most of the CGA has taken place beforehand (24). In essence, the Day Hospital is a “hub” or “command center” to integrate the delivery of CGA services, subspecialty clinics, and preventative healthcare to those most in need.

The Day Hospital may be the ideal location to encourage anticipatory care planning including end-of-life care and cancer survivorship care planning, which help people think about their future health and social care needs. When aligned with CGA, the development of a person-centered care plan to promote this way of thinking about the future in a non-acute care setting enables the older person, their family members, and the multidisciplinary team to address changing needs, complexity, and requirement for support, surveillance, and monitoring from health and social care systems (24). Advanced and personalized care planning is most sustainable when incorporated into routine care in a specialized, dedicated environment where patient trajectories can be predicted and followed (25, 26). Similarly, cancer survivorship care is poorly coordinated in general practice, with little evidence for its integration into routine care (27). Older patients with chronic conditions such as dementia (20) and older cancer survivors (28), whose care needs are markedly different from younger patients could benefit from the CGA, monitoring and advanced care planning delivered in a Day Hospital setting offered in an arguably more appropriate, unhurried and timely manner than in primary or secondary care.

Interventions to target frailty transitions and potentially reverse or prevent onset of frailty may also be best delivered in a Geriatric Day Hospital. A randomized controlled trial that assessed the effectiveness of CGA and subsequent intervention in pre-frail and frail community-dwelling older adults based on the Fried’s criteria found that CGA and subsequent intervention showed a favorable outcome based on frailty status and the Barthel Index of activities of daily living (29). More recently, randomized trial data have shown that targeting pre-frailty using two-staged frailty screening followed by more detailed assessment with tailored multi-factorial interventions may slow progression to frailty (30) and is acceptable to community-dwelling older adults (31). Similarly, programs that promote active and healthy aging that improve outcomes in randomized trials such as the “I am active program” (32) could be coordinated or delivered in Day Hospitals.

The use of innovative ICT solutions to improve care for older adults attending the Day Hospital may represent the next step in re-purposing the construct. There is a growing consensus that these new approaches can drive active and healthy aging (33), and it is argued that the use of ICT in ambulatory care settings (including Geriatric Day Hospitals) could be used to promote this through improved diagnostics, individualized telemedicine, and by enhancing connectivity, social engagement, and continued learning (eHealth literacy) among older adults (34). This use of “silver innovations” to support active aging and healthcare strategies has already proven useful in community-based samples in countries ranging from the Netherlands (35) and Italy (36) to the United States (37) and Japan (38), though these innovations require educational, financial, and policy supports to succeed (38). Although the extent to which ICT can be promoted and implemented in a Day Hospital setting is unknown, its success is likely to be similar to its use in a home care setting (39). The expected shift to greater use of remote monitoring using mobile ICT health technologies is predicted to require more infrastructure (40), particularly for older adults who will require greater support to utilize these services. Traditional services like the Geriatric Day Hospital could be leveraged to this new purpose by providing a “hub” to assist and supervise this for appropriate patients rather than require the building of new and likely commercial infrastructure, which may not have the means or interest to serve this distinct and specialized group. Given that older adults attending a Geriatric Day Hospital in Ireland rated their experience with ICT as limited (41), eHealth literacy would also need to be fostered in this setting. This is echoed by evidence that a supportive environment attuned to the needs of older adults is required for them to effectively use ICT (42).

In a time of limited resources, the Geriatric Day Hospital is as important as ever. Current healthcare systems, under pressure from aging demographics, should re-examine its role and the evidence base for the care it provides, which has arguably not received the attention and recognition that it deserves. Day Hospitals have the potential to evolve and manage the care of older adults with complex care needs across the spectrum from active aging to pre-frailty and from established frailty to end-of-life care. While they should continue to focus on providing CGA, capitalizing on the growing evidence for a frailty syndrome has helped rationalize this limited resource more appropriately (43), Day Hospitals should focus increasingly on providing innovative and proactive, preventative approaches including those that use new mobile ICT technologies to promote healthy aging, address pre-frailty and prevent or reverse frailty at an early stage, before the onset of functional decline (33). Day Hospitals can also be used to promote a system’s wide integrated program of care and education for older adults and healthcare professionals. Thus, although in the future the Day Hospital is likely to remain clinically focused, given its flexibility, it should be able take on new role as a connector “hub” to link primary, secondary, social, and public healthcare; to promote the use of new ICT developments to screen, monitor, and manage the care of community-dwelling older adults; and to advance educational initiatives and eHealth literacy to encourage active and healthy aging well into the twenty-first century.

## Author Contributions

All the authors (ROC, SK, DOS) contributed equally to the planning and writing of the manuscript.

## Conflict of Interest Statement

The authors declare that the research was conducted in the absence of any commercial or financial relationships that could be construed as a potential conflict of interest.
